# Complete chloroplast genome sequence of *Heritiera angustata* (Malvaceae): an endangered plant species

**DOI:** 10.1080/23802359.2017.1422398

**Published:** 2018-02-01

**Authors:** Kun-Kun Zhao, Jian-Hua Wang, Ya-Cheng Cai, Zhi-Xin Zhu, Jordi López-Pujol, Hua-Feng Wang

**Affiliations:** a Hainan Key Laboratory for Sustainable Utilization of Tropical Bioresources, Institute of Tropical Agriculture and Forestry, Hainan University, Haikou, China;; b State Key Laboratory of Biocontrol and Guangdong Provincial Key Laboratory of Plant Resources, School of Life Sciences, Sun Yat-Sen University, Guangzhou, China;; c Botanic Institute of Barcelona (IBB-CSIC-ICUB), Barcelona, Spain

**Keywords:** *Heritiera angustata*, chloroplast genome, illumina sequencing, phylogenetic analysis

## Abstract

*Heritiera angustata* (Malvaceae) is an evergreen tree distributed in the Chinese provinces of Hainan and Yunnan and in Cambodia. In China, it is listed as ‘Endangered’ (EN) *China Red List of Higher Plants*, although it is not protected by law. The complete chloroplast (cp) genome sequence of this threatened species is reported in this study, based on high-throughput sequencing (Illumina). The complete cp genome is 168, 953 bp in length, containing a pair of inverted repeat regions (IRs) of 34,491 bp, a large single copy (LSC) region of 89,054 bp, and a small single copy (SSC) region of 10,917 bp. The cp genome contains 129 genes, consisting of 85 protein-coding genes, 36 tRNA genes and eight rRNA genes. The overall AT content in the cp genome of *H. angustata* is 63.2%. The phylogenetic analyses indicate that there is a close relationship between *H. angustata* and *Firmiana pulcherrima*.

*Heritiera angustata* Pierre (Malvaceae) is an evergreen tree widely distributed in the mountains or near coastal areas in southeast region of Hainan and in Yunnan provinces of China, as well as in Cambodia (Tang et al. 2007). It is listed as ‘Endangered’ (EN) in the *China Red List of Higher Plants* (MEP-CAS 2013). Despite its threatened status, it is not protected by law in China, although some of its populations are included within nature reserves. Genetic diversity is pre-requisite for evolutionary adaptation; thus, preservation of genetic diversity should be a key factor in species’ conservation plans (Hamrick et al. 1991). In order to inform a comprehensive conservation strategy for this threatened species, an improved understanding of its genomics information is urgently needed. Here, the complete chloroplast genome of *H. angustata* (GenBank: this study) is determined and presented, based on the Illumina paired-end sequencing data.

In this study, we sampled a healthy individual of *H. angustata* from Diaoluo Mountain (18.67°N, 109.88°E), which is a National Nature Reserve of Hainan, China. We employed the modified CTAB method (Doyle and Doyle 1987) to extract the total genomic DNA of *H. angustata* from silica gel-dried leaves. A voucher specimen of *H. angustata* (H.-F. Wang B38) was deposited at the Institute of Tropical Agriculture and Forestry, Hainan University (Haikou, China). Whole-genome short-gun sequencing was performed on the Illumina Hiseq 2500 platform, with the 150 bp paired-end sequencing method. After filtering and trimming with NGSQC-Toolkit v2.3.3 (Patel and Jain 2012), the clean reads with an average coverage of 300 × were used to conduct the referenced-based chloroplast genome assembly with the program MITObim v1.8 (Hahn et al. 2013). All genes were annotated with the program of GENEIOUS R8.0.2 (Biomatters Ltd., Auckland, New Zealand). The chloroplast genome of *Theobroma cacao* (HQ336404.2) (Jansen et al. 2011) was used as the reference for assembling and annotation. The annotation was corrected with Dual Organellar Genome Annotator (DOGMA) software (Wyman et al. 2004), and a circular plastid genome map was generated with OGDRAW (Lohse et al. 2013).

The cpDNA of *H. angustata* was a circular molecule of 168,953 bp with a quadripartite structure, containing a pair of inverted repeats (IRs) of 34,491 bp, separated by a large single copy (LSC) region, and a small single copy (SSC) region of 89,054 and 10,917 bp, respectively. It contained 129 genes, including 85 protein-coding genes (76 PCG species), eight ribosomal RNA genes (four rRNA species) and 36 tRNA genes (29 tRNA species). Among these, 13 genes (*trnA-UGC*, *trnI-GAU*, *trnK-UUU*, *trnL-UAA*, *trnV-UAC*, *atpF*, *ndhA*, *ndhB*, *petB*, *petD*, *rpoC1*, *rpl2*, and *rpl16*) harboured a single intron and one gene (*ycf3*) had two introns. The majority of gene species in *H. angustata* occurred as a single copy. The overall AT content of *H. angustata* chloroplast genome was 63.2%, while the corresponding values of the LSC, SSC and IR regions were 65.1%, 68.28% and 59.95%, respectively.

A maximum likelihood (ML) phylogenetic tree of the nine published complete chloroplast genomes of Malvales (plus *H. angustata*) was built with RAxML (Stamatakis 2006), using *Braya humilis* (Brassicaceae, Brassicales) as outgroup (Figure 1). The phylogenetic analysis indicated that all members of Malvales were clustered with a high bootstrap support (BS) value and there was a close relationship between *H. angustata* and *Firmiana pulcherrima*. In this paper, we report the characterization of the complete chloroplast genome of *H. angustata* for the first time, which may provide a useful resource for conservation genetics studies of *H. angustata*, and also for phylogenetic studies of Malvales.

**Figure 1.**
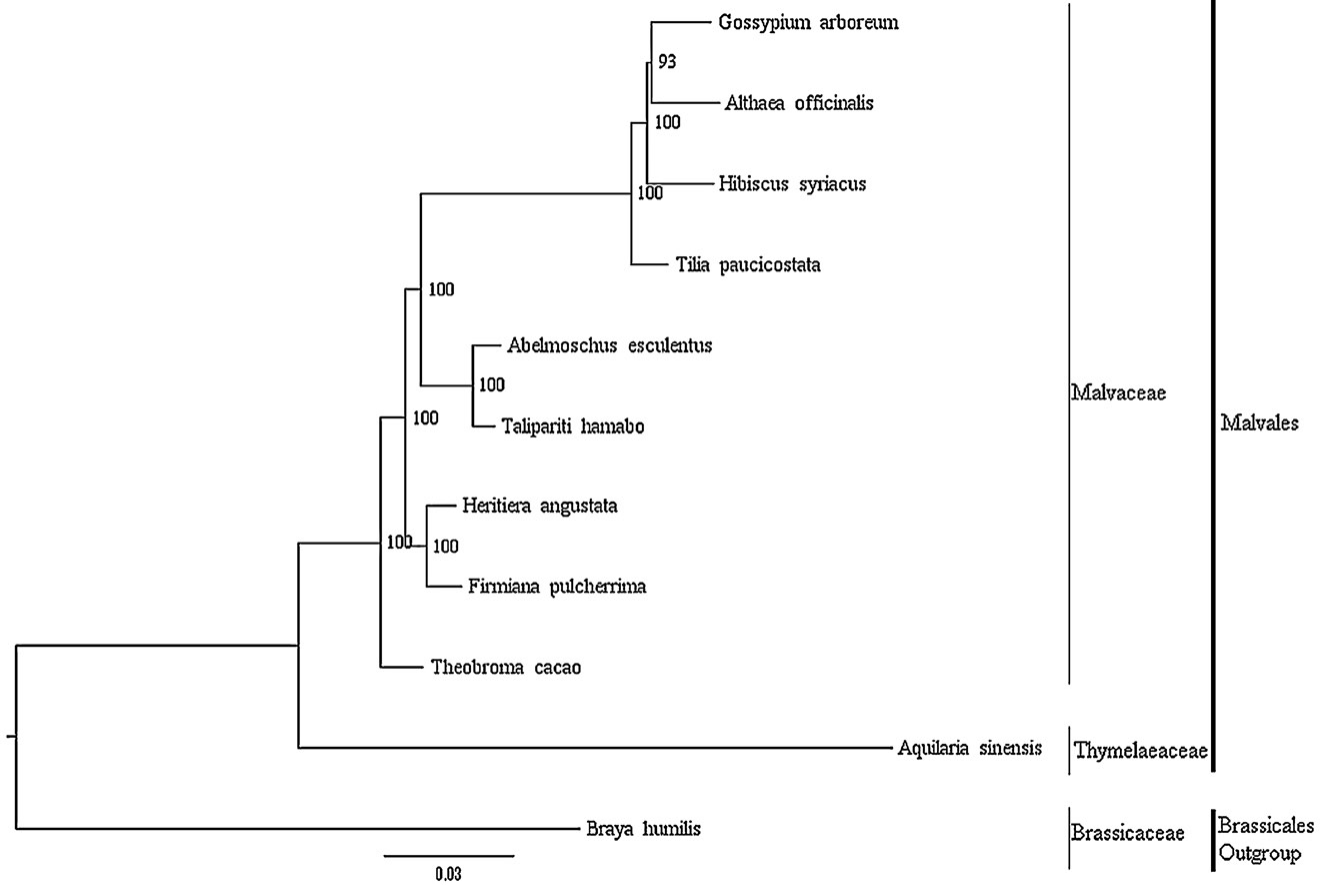
Phylogenetic tree based on 10 complete chloroplast genome sequences of Malvales. Accession numbers:, *Abelmoschus esculentus* NC_035234.1, *Althaea officinalis* NC_034701.1, *Aquilaria sinensis* KT148967.1, *Firmana pulcherrima* MF621982.1, *Gossypium arboreum* HQ325740.1, *H. angustata* (this study), *Hibiscus syriacus* KR259989.1, *Talipariti hamabo* NC_030195.1, *Theobroma cacao* HQ336404.2, *Tilia paucicostata* NC_028591.1; outgroup: *Braya humilis* KY912032.1.

## References

[CIT0001] Doyle JJ, Doyle JL. 1987 A rapid DNA isolation procedure for small quantities of fresh leaf tissue. Phytochem Bull. 19:11–15.

[CIT0002] Hahn C, Bachmann L, Chevreux B. 2013 Reconstructing mitochondrial genomes directly from genomic next-generation sequencing reads-a baiting and iterative mapping approach. Nucleic Acids Res. 41:e129. 23661685 10.1093/nar/gkt371 PMC3711436

[CIT0003] Hamrick JL, Godt MJW, Murawski DA, Loveless MD. 1991 Correlations between species traits and allozyme diversity: implications for conservation biology In: Falk DA, Holsinger KE, editors. Genetics and conservation of rare plants. New York: Oxford University Press; p. 75–86.

[CIT0004] Jansen RK, Saski C, Lee S-B, Hansen AK, Daniell H. 2011 Complete plastid genome sequences of three rosids (*Castanea*, *Prunus*, *Theobroma*): evidence for at least two independent transfers of *rpl22* to the nucleus. Mol Biol Evol. 28:835–847. 20935065 10.1093/molbev/msq261 PMC3108605

[CIT0005] Lohse M, Drechsel O, Kahlau S, Bock R. 2013 OrganellarGenome-DRAW: a suite of tools for generating physical maps of plastid and mitochondrial genomes and visualizing expression data sets. Nucleic Acids Res. 41:W575–W581. 23609545 10.1093/nar/gkt289 PMC3692101

[CIT0006] Ministry of Environmental Protection–Chinese Academy of Sciences (MEP-CAS) 2013. China red list of higher plants: evaluation’s report. Ministry of Environmental Protection of the People’s Republic of China & Chinese Academy of Sciences, Beijing [in Chinese].

[CIT0007] Patel RK, Jain M. 2012 NGS QC Toolkit: a toolkit for quality control of next generation sequencing data. PLoS One. 7:e30619. 22312429 10.1371/journal.pone.0030619 PMC3270013

[CIT0008] Stamatakis A. 2006 RAxML-VI-HPC: maximum likelihood-based phylogenetic analyses with thousands of taxa and mixed models. Bioinformatics. 22:2688–2690. 16928733 10.1093/bioinformatics/btl446

[CIT0009] Tang Y, Gilbert MG, Dorr LJ. 2007 Sterculiaceae In: Wu ZY, Raven PH, Hong DY, editors. Flora of China. Vol. 12 (Hippocastanaceae through Theaceae). Beijing, St. Louis: Science Press and Missouri Botanical Garden Press; p. 302–330.

[CIT0010] Wyman SK, Jansen RK, Boore JL. 2004 Automatic annotation of organellar genomes with DOGMA. Bioinformatics. 20:3252–3255. 15180927 10.1093/bioinformatics/bth352

